# Improved efficiency of the new Mirus™ system challenged by ventilatory settings—a bench study

**DOI:** 10.1007/s10877-025-01344-5

**Published:** 2025-08-23

**Authors:** Frederic Albrecht, Claudia Schirra, Kathrin Scheffler, Thomas Volk, Andreas Meiser

**Affiliations:** 1 Department of Anesthesiology, Intensive Care and Pain Therapy, Saarland University Medical Center and Saarland University Faculty of Medicine, Homburg, Germany; 2 Outcomes Research Consortium®, Houston, TX USA

**Keywords:** Volatile anaesthetics, Inhaled sedation, Mirus, Isoflurane, Sevoflurane

## Abstract

The Mirus™ system (Technologie Institut Medizin GmbH, Germany) enables target-controlled delivery of volatile anaesthetics with open system ventilators. Its interface, positioned between Y-piece and the patient, injects anaesthetic vapor during inspiration, while adsorbing anaesthetic and resupplying it during the next inspiration. A newly introduced interface (Lisa-44) was evaluated under body temperature pressure saturated and normocapnic conditions using a test lung. Volume-controlled ventilation was applied with a 500 mL tidal volume, 10 bpm respiratory rate, and inspiratory flow (IF) times of 2.5 s and 0.5 s. Isoflurane (sevoflurane) target concentrations were stepwise increased to 1.5 MAC and then decreased. An external gas monitor recorded real-time anaesthetic concentrations. End-tidal concentrations (EC) and area under the curve (AUC) were analyzed for each breath. Accuracy and precision were assessed using Bland-Altman plots. Consumption was calculated and compared to historical controls. Mirus EC measurements correlated well with external gas monitor readings. ECs fluctuated around targets, with higher targets requiring more frequent injections and larger AUCs. Anaesthetic consumption was lower than historical controls and comparable to the Sedaconda ACD. At 0.3–0.5 MAC, hourly isoflurane consumption ranged from 1.0 to 1.7 mL (sevoflurane: 3.8–6.1 mL). However, very short IF times significantly increased consumption, reaching 43 mL sevoflurane at an EC of 2.6 vol%, with prolonged injections extending into the expiration phase. The new system demonstrates high accuracy, precision, and improved efficiency, suggesting reduced anaesthetic consumption in clinical use. However, high concentrations combined with very short IF times substantially increase consumption, indicating potential limitations.

## Introduction

 Critically ill patients frequently require sedation to tolerate endotracheal intubation, mechanical ventilation or other interventions during their stay in the intensive care unit (ICU). The use of volatile anaesthetics for ICU sedation has increased recently and is recommended by guidelines [1–4]. Benefits of inhaled sedation include faster awakening, precise drug concentration monitoring, opioid sparing effects, and preservation of spontaneous breathing activity [5–8]. Delivering volatile anaesthetics via conventional ICU ventilators requires special medical devices positioned between the Y-piece and the patient [9].

The MIRUS™ (Technologie Institut Medizin GmbH, Koblenz, Germany) consists of a control unit and an interface. The Mirus Controller, available since 2013 and holding a current CE certificate from January 2023 [10], measures ventilatory parameters and gas concentrations enabling target-controlled delivery of volatile anaesthetics. It supports isoflurane and sevoflurane, with an interface compatible with both agents. Similar to available reflection devices, the interface contains an anaesthetic carbon reflector that absorbs exhaled volatile anaesthetics and releases them during the subsequent inspiration, reducing consumption relative to open systems [11–13].

A new Mirus interface version, LISA 44 (Technologie Institut Medizin GmbH), recently received regulatory approval [14] and is commercially available for isoflurane and sevoflurane. It features reduced dead space (44 mL vs. 50 mL in the previous model) and compatibility with various commercially available heat moisture exchangers (HMEs), which generally have smaller internal volumes than the prior integrated HME adding 50 mL dead space [15].

According to the manufacturer, the revised system has an improved reflection efficiency. We aimed to verify this claim by comparing anaesthetic consumption at different target concentrations with published data from the previous version and alternative devices. Additionally, we re-assessed the accuracy and precision of the measured end-tidal anaesthetic concentration (EC), its agreement with the target concentration and described the updated target control algorithm.

As gaseous anaesthetic is injected into the breathing gas at the beginning of inspiration, we hypothesized that anaesthetic consumption might also depend on ventilatory settings. Specifically, we investigated whether consumption increases under conditions of very short inspiratory flow times seen in spontaneously breathing patients. Using a setup similar to our previous study [13], we re-evaluated the new system on the bench, ensuring normocapnia and body temperature pressure saturated (BTPS) conditions throughout.

## Methods

### Experimental setup

**Fig. 1:**
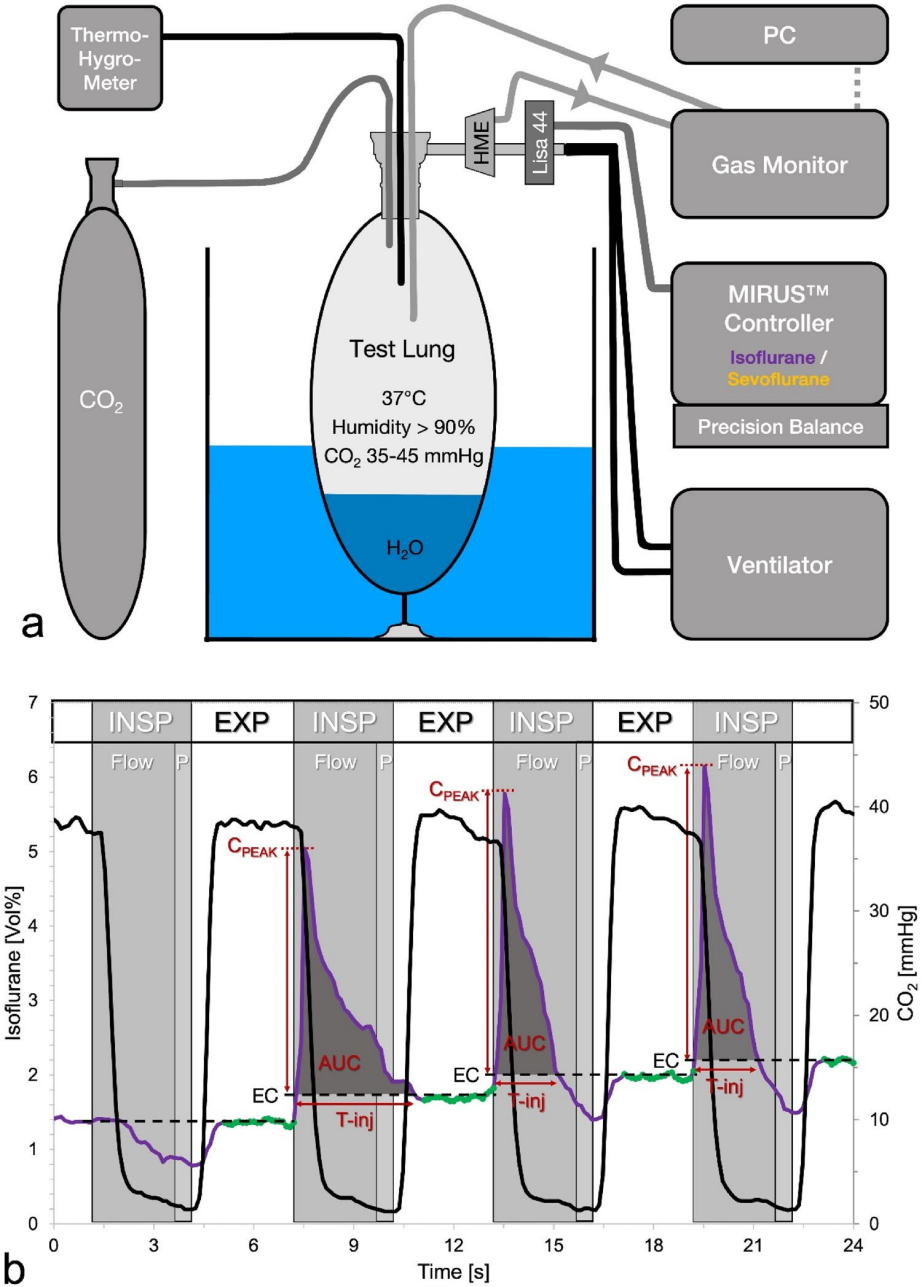
**a** Experimental setup. To achieve body temperature pressure conditions, a test lung filled with 100 mL distilled water was immersed in a heated aquarium. Temperature and humidity were continuously monitored. Carbon dioxide (CO_2_) was introduced to achieve normocapnic conditions. The Mirus™ controller, connected to its interface LISA-44, measured ventilatory parameters and gas concentrations while delivering isoflurane or sevoflurane at target concentrations. A side stream gas monitor was connected to the heat moisture exchanger (HME). Real time gas concentrations were stored at 100 msec intervals on a computer (PC). **b** Volatile anaesthetic and CO2 concentrations over four respiratory cycles. All data were recorded by an external gas monitor at 100 msec intervals. Example tracing with isoflurane at a target concentration of 1.68 Vol%. Volume controlled ventilation with an inspiratory flow time of 2.5 s (Flow), an inspiratory pause of 0.5 s (P) and an expiration phase of 3 s (Exp). Note that the onset of inspiration (INSP) precedes the decline of the CO2 tracing because CO2 rich expired gas retained inside LISA 44 is passing the gas sampling port at early inspiration. Consequently, only 20 (highlighted in green) out of 30 isoflurane concentration measurements during elevated CO2 levels were averaged to calculate the endtidal anaesthetic concentrations (EC, dashed lines). Saturated isoflurane vapour was injected during the last three respiratory cycles shown. Peak concentrations (Cpeak), injection time (T-inj) and area under the curve (AUC) for each injection were calculated for further analysis. Note that the first anaesthetic injection extends beyond the inspiratory flow time, potentially reducing its efficiency. Additionally, during inspiration, CO2 concentration does not decrease to zero because of minor CO2 reflection by the reflector

An Evita IV ventilator (Dräger Medical, Lübeck, Germany) was connected via appropriate ventilatory hoses (Coaxial breathing circuit set, Hamilton Media, Bonaduz, Switzerland) to a test lung (3 L manual breathing bag for Zeus; Dräger Medical) (Fig. 1a). The test lung was filled with 100 mL of distilled water and immersed in heated water to achieve BTPS conditions. A continuous flow of CO_2_ was delivered from a gas cylinder (Air Liquide Deutschland GmbH, Düsseldorf, Germany) equipped with a pressure regulator (Swiss optio basic regflow, GLOOR AG, Burgdorf, Switzerland). The CO_2_ was administered via an oxygen delivery tube (Teleflex, Morrisville, USA) through the bronchoscopy port into the test lung. The flow rate was adjusted to maintain normocapnic conditions, ensuring an end-tidal CO_2_ concentration of 35 to 45 mmHg.

The Mirus control unit was positioned on a precision scale (TC20K-HB, G & G GmbH, Kaarst, Germany) and connected to its interface LISA 44. The interface and a standard HME (Humid-Vent^®^ Filter Compact, Teleflex, Morrisville, USA) were placed between the Y-piece and the test lung.

A side stream gas monitor (Vamos^®^, Dräger Medical) was connected to the gas sampling port of the HME. To prevent anaesthetic gas loss, the sampled gas was reintroduced into the test lung. Data from the gas monitor were transferred to a notebook computer.

### Ventilatory settings

Volume-controlled ventilation with constant flow was set, with an oxygen fraction of 0.21, a positive end-expiratory pressure of 3 hPa, a respiratory rate of 10 breath per minute, and a tidal volume of 500 mL, maintaining an inspiration to expiration ratio of 1:1 for all experiments. In some experiments, the inspiratory flow (IF) was set at 12 L/min, resulting in a long IF time of 2.5 s, while in others, the IF was set to 60 L/min, reducing the IF time to 0.5 s.

### Target control settings

As the Mirus Controller is specific for either isoflurane or sevoflurane, two separate control units were used. After inputting a patient age of 40 years, the Mirus assumed a minimal alveolar concentration (MAC) of 1.2 Vol% for isoflurane and 1.9 Vol% for sevoflurane. The target concentration was then set as a fraction of MAC.

After a thirty-minute equilibration period to establish BTPS conditions and normocapnia, the target concentration was initially set at 0.2 MAC and subsequently increased every 10 min by 0.2 MAC until 1.4 MAC. The target was then increased to 1.5 MAC (the maximum target) for 20 min, before being reduced to 1.4 MAC and then decreased by 0.2 MAC every ten minutes until reaching 0.0 MAC.

Four independent experiments were conducted on different days, comparing long and short IF times and using isoflurane and sevoflurane, respectively.

### Data recording

Real time anaesthetic gas concentrations were recorded in % volume to the second decimal point and stored at 100 msec intervals on the notebook computer. CO_2_ readings were recorded simultaneously. Consequently, for each experiment, 160 min of data were recorded, corresponding to 1,600 single breaths or 96,000 anaesthetic and 96,000 CO_2_ gas measurements.

After each experiment, the internal storage of the Mirus control unit was read out yielding breath to breath gas concentration and ventilatory data as well as the (decreasing) volume of liquid anaesthetic still contained in the control unit.

Manuel readings from the precision scale at the beginning, end, and occasionally during the experiments were used to verify the anaesthetic consumption data recorded by the Mirus system.

### Data evaluation

Gas measurements from the Vamos gas monitor were transferred to an Excel sheet (Microsoft Corporation, Redmond, USA) where each row corresponded to one 100 msec measurement interval. All CO_2_ concentrations exceeding 34 mmHg were marked to roughly identify expiration. During the expected 3 s expiration phase, 30 anaesthetic gas measurements were anticipated. However, the final CO_2_ signals were associated with inspiratory flow, as CO_2_ rich expired gas remained in the Mirus interface at the end of expiration and was subsequently pushed toward the patient and the gas sampling port of the HME during the next inspiration [16]. In fact, inspiratory injections of anaesthetic could be detected in parallel to increased CO_2_ concentrations. To account for this, only 20 anaesthetic gas measurements beginning 0.2 s after the first CO_2_ concentration above 34 mmHg were averaged and interpreted as endtidal anaesthetic concentration (EC) (Fig. 1b).

During some breaths, anaesthetic injections resulted in concentration peaks at early inspiration. To quantify these injections, the EC of the subsequent breath was subtracted from these real-time concentration peaks. The number of positive values, divided by ten, represented the peak duration in seconds. The sum of these positive values corresponds to the area under the curve of each peak. Dividing this sum by 10 will yield % volume times second as measuring unit (Fig. 1b).

Data retrieved from the internal storage of the Mirus were also transferred to an Excel sheet. From the filling volume of the anaesthetic reservoir, the anaesthetic consumption during each 10-minute period with constant concentration target was calculated. The consumed volumes during the increasing and decreasing series were averaged and extrapolated to an hourly rate.

Reflection efficiencies (REs) were calculated as previously described [17]:

$$RE{\text{ }}\left[ \% \right]{\text{ }} = {\text{ }}100{\text{ }}*{\text{ }}(1 - \left( {100{\text{ }}*{\text{ }}IR{\text{ }}*{\text{ }}F{\text{ }}/{\text{ }}\left( {60{\text{ }}*{\text{ }}EC{\text{ }}*{\text{ }}VT{\text{ }}*{\text{ }}RR} \right)} \right)$$

where IR = infusion rate [ml/hour], F = factor for converting liquid anaesthetic to vapour (isoflurane: 200 mL/mL, sevoflurane: 193 mL/mL), EC = endtidal concentration [vol%], VT = tidal volume [mL], RR = respiratory rate [bpm].

### Statistics

To compare EC measurements obtained from the Vamos gas monitor and the Mirus Controller, Bland-Altman diagrams were generated [18]. ECs measured by Vamos and Mirus, the target concentrations, and the mean ECs for each target as measured by Vamos were plotted against time.

Anaesthetic consumption values from all four experiments were plotted against the EC as measured by Vamos and fitted with regression lines. To compare anaesthetic consumption among the four experiments, multiple linear regression was conducted with IBM SPSS Statistics 30 (IBM Corporation, Armonk, USA). For comparison between isoflurane with sevoflurane, the last three target concentrations of sevoflurane were excluded to account for higher target concentrations when using sevoflurane, where a disproportionately increased consumption could be expected.

To compare anaesthetic consumption with historical data, reflection efficiencies (RE) were plotted against the volume of anaesthetic vapour contained in a single exhaled breath.

All analyses are descriptive in nature. A p value below 0.05 was considered significant. Corrections for multiple testing were not applied.

## Results

**Fig. 2:**
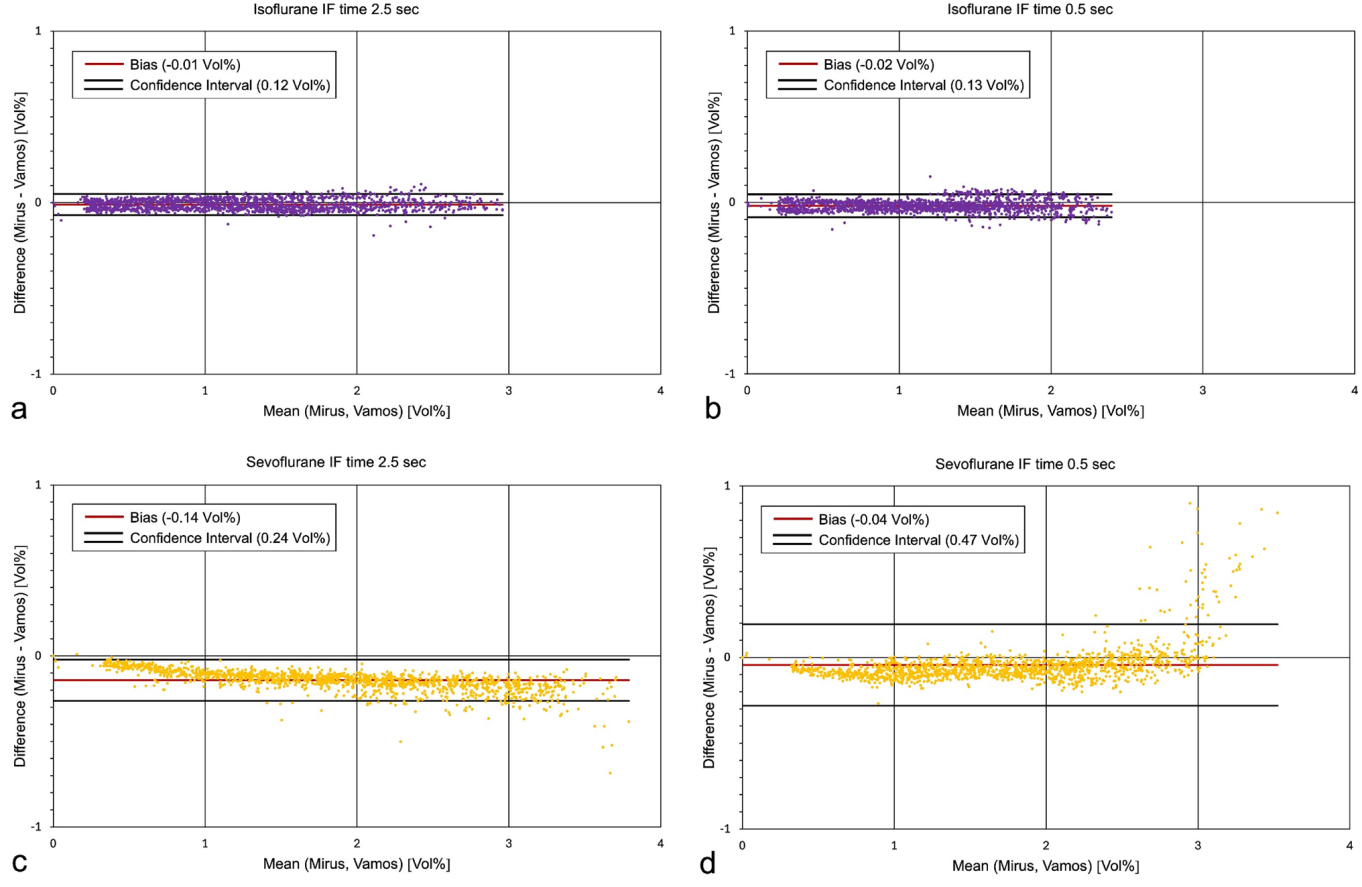
Bland-Altman diagrams illustrate the agreement between the endtidal anaesthetic concentrations measured by Mirus™ and the Vamos^®^ gas monitor (a&b: isoflurane (violet), c&d: sevoflurane (yellow)). a&c: ventilation mode with long inspiratory flow (IF) time of 2.5 s. b&d: ventilation mode with short IF time of 0.5 s. Note that with the long IF time, higher concentrations are reached (X-axis). Additionally, for sevoflurane with the short IF time, measurement differences increase at high concentrations, suggesting an overestimation by Mirus

Measurements of the ECs obtained from the Mirus™ control unit, and the Vamos^®^ gas monitor demonstrated good agreement in Bland Altman diagrams, with small biases (accuracy) and narrow limits of agreement (precision), except in the last experiment at high sevoflurane concentrations, where EC measurements by Mirus exceeded those recorded by Vamos considerably (Fig. 2).

**Fig. 3:**
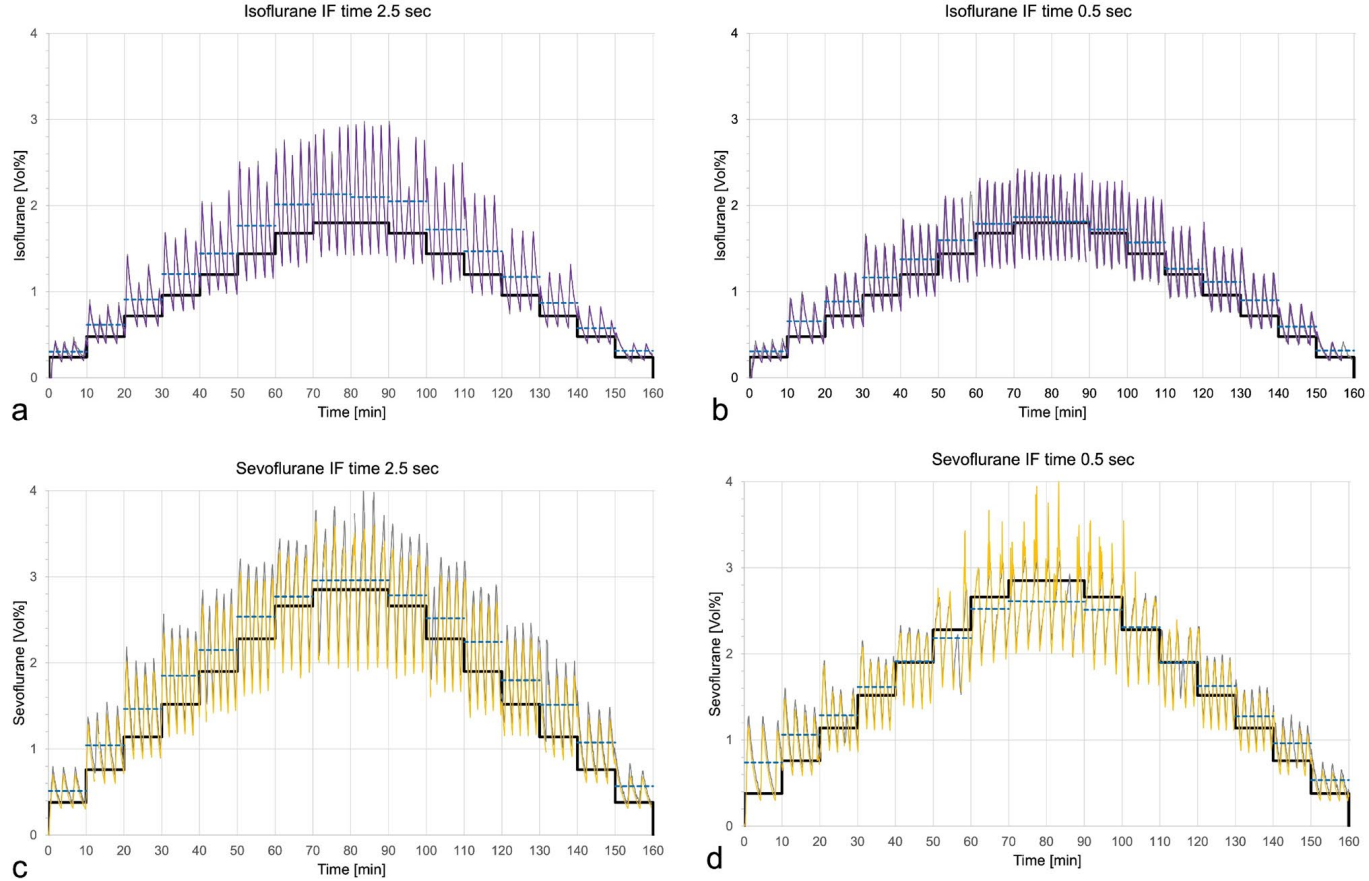
Endtidal concentrations (EC) of isoflurane (a&b) and sevoflurane (c&d), as measured by the Vamos gas monitor (grey lines) and the Mirus™ (overlying violet or yellow lines), are shown over a 160-minute period for each experiment. The target concentration (black line) was increased every 10 min by 0.2 minimal alveolar concentration equivalents (MAC; 1.2 Vol% for isoflurane and 1.9 volume % for sevoflurane) up to a maximum adjustable target of 1.5 MAC, after which it was gradually decreased. Dashed blue lines represent the mean EC (Vamos) for each target. a&c: ventilation mode with long inspiratory flow (IF) time of 2.5 s. b&d: ventilation mode with short IF time of only 0.5 s. Note that at the highest target in panel d, ECs measured by Mirus are overestimated, while the target is not reached (dashed blue line is below black line)

ECs measured by Vamos are swinging around the target concentrations with mean values consistently exceeding the target, except at high target concentrations during the middle phase of the last experiment (Fig. 3).

**Fig. 4:**
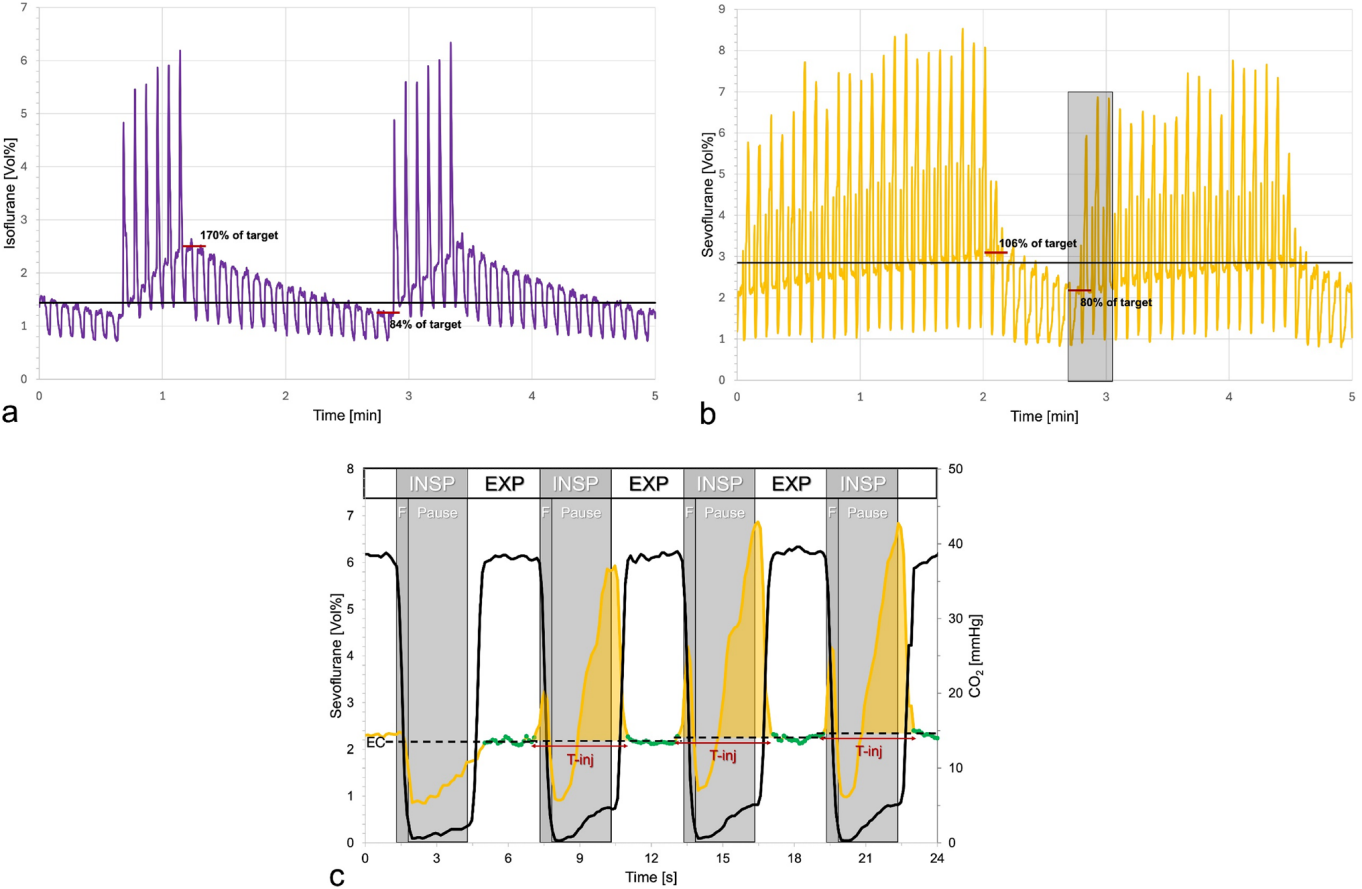
Short sequences of real time anaesthetic concentrations measured by the Vamos gas monitor. **a**: Isoflurane at a 1.44 Vol% target concentration (1.2 minimal alveolar concentration equivalent, black line) under volume-controlled ventilation with a long inspiratory flow time (2.5 s). A series of 6 injections of saturated steam of isoflurane during the inspiratory phase results in an increase of endtidal concentrations up to 70% above the target. Following this, endtidal concentrations decrease to 16% below the target, after which another series of six injections is administered. **b**: Sevoflurane at 2.85 Vol% target concentration (1.5 minimal alveolar concentration equivalent, black line) under volume-controlled ventilation with a short inspiratory flow time (0.5 s). A total of 23 anaesthetic injections are required to raise the endtidal concentrations up to 6% above the target. Subsequently, endtidal concentrations drop to 20% below target, when another series of 20 injections starts. **c**: Enlarged view (grey shaded area) from panel b. Sevoflurane injections exhibit a biphasic peak. The first portion of anaesthetic is injected around the start of the inspiration (INSP, first peak), and is blown away quickly by the high and short-lived inspiratory flow (F) of the ventilator. The injection time (T-inj) is too long and continues into the inspiratory pause. A high concentration builds up in the Mirus interface (second peak), which is then pushed against the reflector during the subsequent expiration (EXP), overloading the capacity of the reflector. Note that the endtidal concentrations (EC, dashed lines) increase only minimally. As a result, anaesthetic delivery becomes less efficient compared to the sequence shown in Fig. 4a

Over time, ECs gradually declined below the target level until a series of anaesthetic vapor injections was administered (Fig. 4). Depending on the IF time, target concentration, and volatile anaesthetic used, the number of injections varied from 6 to 26, with peak durations ranging from 0.4 to 4.3 s. The resulting AUCs ranged from 0.4 to 5.4 Vol%*sec, with a mean of 2.7 Vol%*sec, which was sufficient to restore ECs above the target level.

At high targets with short IF times, anaesthetic injections exhibited a biphasic peak pattern, with the last peak occurring during the inspiratory pause and extending into the subsequent expiration. As a result, a high concentration of anaesthetic will be pushed against the reflector, exceeding its capacity and leading to a markedly reduced RE.

**Fig. 5:**
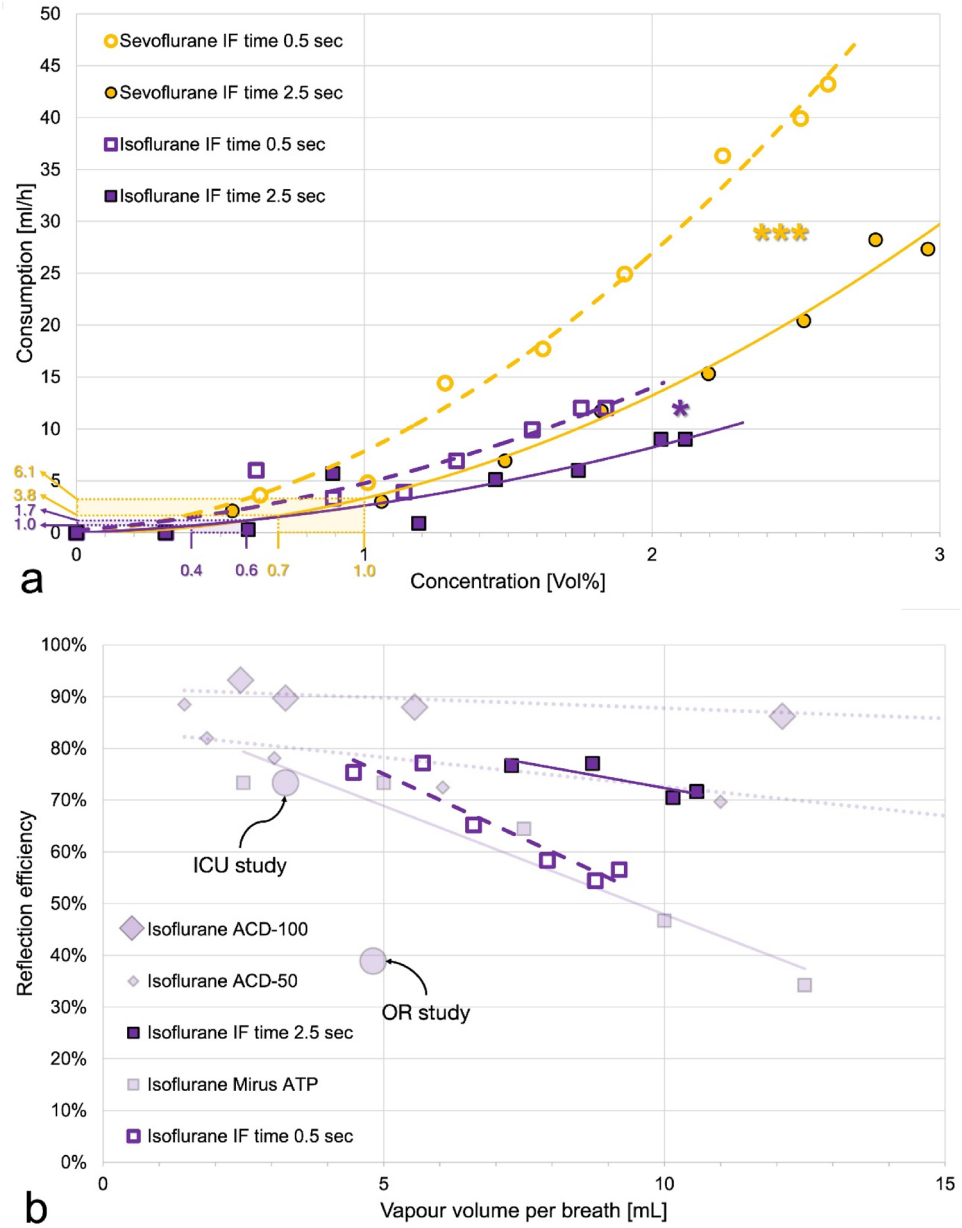
**a** Isoflurane or sevoflurane consumption plotted over the mean endtidal concentration achieved for each target of the four experiments. Polynomial regression lines with increasing slopes gave the best fits. For the clinical range of concentrations used (0.4 to 0.6 Vol% for isoflurane, 0.7 to 1.0 vol% for sevoflurane), anaesthetic consumptions can be calculated as 1.0 to 1.7 mL isoflurane or 3.8 to 6.1 mL sevoflurane per hour. Note that in clinical practice consumption will be higher due to patient uptake and leaks (closed endotracheal suctioning, tube disconnections, gas sampling for monitoring etc.). Multiple linear regression showed significant differences between the ventilation modes for isoflurane (*, *P* = 0.012) and for sevoflurane (***, *P* < 0.001) as well as between sevoflurane and isoflurane for the short inspiratory flow (IF) time (*P* = 0.022) but not for the long IF time. For the latter comparisons, the last three sevoflurane targets were omitted because high concentrations are known to increase consumption disproportionately. **b** Reflection efficiencies are plotted over the vapour volume per exhaled breath. Data from the current study are represented in bright colours, historical data are shown in pale colours for comparison. Because of wide scattering, data for the lower targets are excluded; only isoflurane data are presented. Historical data for Sedaconda-ACD 50 (small diamonds) and Sedaconda ACD-100 (large diamonds) were obtained under comparable bench conditions (similar ventilation, normocapnic, body temperature pressure conditions) [17]. Historical data for Mirus (small pale squares) were also measured with similar ventilation parameters and with carbon dioxide, but under dry laboratory conditions (ambient temperature pressure dry) [20], known to increase (!) reflection efficiency. Circles represent mean values from two clinical studies using Mirus in the intensive care unit (ICU) [22] and in the operating room (OR) [23]

Higher ECs were associated with a disproportionate increase in anaesthetic consumption, as shown by the nonlinear regression lines in Fig. 5a. For both anaesthetics, consumption was significantly higher when short IF times where applied.

The RE of the new Mirus system for isoflurane with the short IF time was comparable to historical laboratory data of the older system obtained under ambient temperature pressure dry (ATPD) conditions with CO_2_. However, with the long IF time, RE was substantially higher and comparable to historical laboratory data from the Sedaconda-ACD-50, measured under similar conditions (BTPS plus CO_2_, Fig. 5b).

## Discussion

In this initial study evaluating the new Mirus system on the bench, we observed low anaesthetic consumptions and consequently improved efficiency compared to its predecessor. In the clinical concentration range, hourly isoflurane consumption ranged from 1 to 2 millilitres, while for sevoflurane it ranged from 4 to 6 millilitres.

The Mirus system injects saturated anaesthetic vapour during early inspiration. This may facilitate quicker attainment of the target concentration and enhance system efficiency. However, when high target concentrations are aimed for, high volumes of anaesthetic vapour must be delivered within a short time. Our experiments demonstrated, that when very short IF times are used, there is insufficient time for the complete injection of anaesthetic vapour (see the second late spike in Fig. 4c). Consequently, during the inspiratory pause, a cloud of anaesthetic vapour accumulates within the Mirus interface and the HME. During the subsequent expiration, this cloud is pushed against the anaesthetic reflector, potentially overwhelming its capacity. This effect was particularly evident in the middle of our last experiment, when high sevoflurane concentrations were combined with a very short IF time (Fig. 3d). This configuration led to a significant increase in anaesthetic consumption (Fig. 5a). In addition, the cloud did not disappear completely during expiration, resulting in falsely elevated EC measurements (Fig. 2d). Notably, only in this last experiment, the means of the EC measurements were slightly below the target concentrations (Fig. 3d).

In older studies [24; 25], as well as during the Covid pandemic [26], anaesthesia circuits were used in the ICU. Through rebreathing with reduced fresh gas flows, a similarly efficient use of volatile anesthetics can be achieved as with reflection. One group proposed two circuits interconnected by an anaesthetic reflector with carbon dioxide absorber and in-line vaporizer placed in the second circuit near the patient to combine advantages of both principles [27]. With this technique, sevoflurane consumption in pigs weighing 50 kg was only 3.6 mL/hour. Today a passive reflection system, the Sedaconda^®^ ACD, formerly called AnaConDa [11], is most commonly used for inhaled sedation [8]. With this system, liquid anaesthetic is continuously administered via a syringe pump into a hollow porous rod evaporating on its surface.

Reflection efficiency (RE) can be defined as the percentage of exhaled anaesthetic molecules that are resupplied to the patient during the subsequent inspiration. Under steady-state-conditions, RE can be calculated from the amount of anaesthetic consumed [17]. Unlike anaesthetic consumption, RE is independent of the minute ventilation, which allows the comparison of different anaesthetic reflectors under various conditions. RE typically decreases with the volume of anaesthetic vapour contained in one breath 

 (Fig. 5b), i.e. with the product of the EC times the tidal volume.

A bench study evaluating the older Mirus system under ATPD conditions showed even lower REs for isoflurane [20] compared to our second experiment when the system was challenged with very short IF times (Fig. 5b). This is unexpected since REs are typically higher under ATPD than BTPS conditions [17]. In our experiments with long IF times, RE values matched those of Sedaconda ACD-50 under similar lab conditions [17]. While the larger Sedaconda ACD-100 performed better [17], its use is limited today due to its significant dead space [21].

In a clinical study evaluating the older Mirus system in the ICU, REs were slightly above 70% with a median isoflurane consumption of 4 mL/hour [22]. In contrast, another study in the operation room, using higher tidal volumes and much higher anaesthetic concentrations, showed REs around 40% with an hourly consumption of 11.2 mL isoflurane [23].

Improving the efficiency of reflection devices seems crucial in view of climate change. The global warming potential of 1 g of isoflurane corresponds to 1.9 kg carbon dioxide over a 20 years period [28]. Based on the data, using the updated Mirus device instead of the previous version could allow for a reduction of up to 2 mL isoflurane per hour. This would amount to savings of 139 kg carbon dioxide equivalents per patient day. In addition to these substantial greenhouse gas reductions, an equivalent decrease in cost and environmental pollution with polyfluorinated alkylic substances is also achieved.

### Limitations

We measured anaesthetic consumption under laboratory conditions using BTPS and normocapnic conditions, which are comparable to clinical conditions at the bedside. However, in clinical practice, anaesthetic consumption is expected to be higher due to patient uptake, which decreases rapidly during the initial hours, as well as leaks caused by closed endotracheal suctioning, tube disconnections, gas sampling for monitoring, and other factors.

Consumption was evaluated during short intervals of 10 min which may lead to imprecise results. Especially, for the lower targets, consumption scattered around the regression lines. We therefore omitted these values in the comparison of the RE with historical controls.

Moreover, we did not measure during steady state conditions but while in- or decreasing the target concentrations. However, the capacity of the test lung is small and its walls do not take up anaesthetic. By averaging wash in and wash out periods of the same target, bias should be excluded.

The test lung was ventilated in volume-controlled mode, which differs from pressure-controlled or pressure-supported modes typically used in the ICU. Volume-control was chosen to align with previous studies and to ensure constant tidal volumes, which were crucial for our calculations. This also allowed us to define inspiratory flow times, which we expected to have an influence on RE. In this study, standardized ventilation settings were applied, and the tidal volume was set to 500 ml to facilitate comparison with existing literature. By considering the reflection efficiency, the results can be extrapolated to other ventilation settings.

Additionally, we did not directly compare the new Mirus system with its predecessor, as consumable materials are no longer commercially available, nor did we compare with the Sedaconda ACD. Furthermore, the extend of carbon dioxide reflection by the new device was not examined in this study.

Future studies should focus on direct comparison of the new Mirus system with the Sedaconda ACD-50 or ACD-100, both in laboratory and clinical studies. Anaesthetic consumption should be assessed under various ventilatory settings, with particular attention to tidal volume, as it can have a direct effect on reflection efficiency. Additionally, handling and safety issues, as well as wash in times, should be evaluated in clinical studies.

## Conclusion

The new Mirus system enables target-controlled inhaled sedation with high precision. Compared to its predecessor, consumption of volatile anaesthetics is substantially decreased. However, very high concentrations combined with short inspiratory flow times may push the system to its limits and increase consumption exponentially. Reduced consumption seems crucial in view of climate change and will have to be confirmed in clinical studies.

## Data Availability

Data can be sent from the corresponding author upon reasonable request.

## Abbreviations

ACD: Anaesthetic conserving device
ATPD: Ambient temperature pressure dry
AUC: Area under the curve
bpm: Breath per minute
BTPS: Body temperature pressure saturated
CE: Conformité Européenne
CO2: Carbon dioxide
EC: Endtidal concentration
EXP: Expiration
F: Factor for converting liquid anaesthetic to vapour
HME: Heat moisture exchanger
ICU: Intensive care unit
IF: Inspiratory flow
INSP: Inspiration
IR: Infusion rate
L: Litre
MAC: Minimal Alveolar concentration
mL: millilitre
msec: milliseconds
mmHg: millimeter mercury
Min: Minutes
OR: Operating room
RR: Respiratory rate
sec: Seconds
T-Inj: Injection time
TM: Trademark
VT: Tidal volume
hPa: Hectopascal
